# Rapid and simple detection of *Chlamydia trachomatis* and *Neisseria gonorrhoeae* using the EasyNAT CT/NG assay based on cross-priming amplification

**DOI:** 10.1007/s10096-025-05218-1

**Published:** 2025-07-22

**Authors:** Qing Wu, Hongxiang Tu, Yingjie Dai, Yumin Wang, Lijuan Hu

**Affiliations:** Department of Laboratory Medicine, Key Laboratory of Clinical Laboratory Diagnosis and Translational Research of Zhejiang Province, The First Affiliated Hospital of Wenzhou Medical University, Wenzhou, China

**Keywords:** *Chlamydia trachomatis*, *Neisseria gonorrhoeae*, Cross-priming amplification, EasyNAT CT/NG assay, Point-of-care testing

## Abstract

**Purpose:**

*Chlamydia trachomatis* (CT) and *Neisseria gonorrhoeae* (NG) rank among the most common sexually transmitted pathogens. Rapid screening and detection of these bacteria are essential to reduce sequelae and prevent transmission. This study evaluated the efficacy of the EasyNAT CT/NG assay, which utilizes cross-priming amplification (CPA) technique for the rapid and simultaneous detection of CT and NG in diverse reproductive tract specimens, achieving diagnosis within 30 min.

**Methods:**

The clinical performance of the EasyNAT CT/NG assay in detecting CT and NG was assessed using 198 clinical samples, with results compared to those of conventional in-house Real-Time PCR to determine concordance. Sensitivity was measured using serial dilutions of quantified plasmids and specificity was evaluated by incorporating DNA from 18 common STI pathogens. The assay’s suitability as a point-of-care testing (POCT) tool was evaluated with the criteria outlined in Target Product Profiles (TPPs).

**Results:**

The EasyNAT CT/NG assay demonstrated high concordance with Real-Time PCR, with rates of 98.5% for CT and 99.0% for NG. Concordance in urine samples reached 98.6% for CT and 100% for NG, while cervical swabs showed both 97.7% for CT and NG; vaginal and urethral swabs achieved 100% for both pathogens. Among the 198 samples, one urine specimen tested negative for CT by Real-Time PCR but positive by the EasyNAT CT/NG assay, a positive result confirmed by the Cepheid Xpert CT/NG assay. Two cervical swabs, negative for CT and NG by Real-Time PCR, yielded invalid results with the EasyNAT CT/NG assay but were confirmed negative or CT and NG by the Cepheid Xpert CT/NG assay. The EasyNAT CT/NG assay reliably detected CT and NG in turbid specimens, though it may fail with severely hemolytic samples. Its detection limit was 400 copies/mL, with no cross-reactivity observed across 18 other pathogens.

**Conclusion:**

The EasyNAT CT/NG assay offers rapid, sensitive, and specific detection of CT and NG, proving valuable for infection screening and early diagnosis. It shows promise as a rapid POCT method.

## Introduction

Sexually transmitted infections (STIs) represent one of the most prevalent categories of acute infectious diseases and continue to present significant public health challenges, with a global increase in incidence [1]. *Chlamydia trachomatis* (CT) and *Neisseria gonorrhoeae* (NG) rank among the most common STIs, with the World Health Organization (WHO) documenting approximately 129 million new CT cases and 82 million new NG cases in 2020 [2]. These infections are often asymptomatic [3] – [4], leading to delayed diagnosis and progression to chronic conditions. Consequently, CT and NG can cause severe reproductive complications, such as epididymitis, salpingitis, pelvic inflammatory disease, ectopic pregnancy, and infertility, particularly in women [5–7]. Furthermore, antibiotic resistance is widespread in NG, complicating treatment and control strategies [8–10]. Thus, early detection and diagnosis are crucial for preventing the spread of sexually transmitted infections.

Current CDC guidelines recommend detecting CT and NG using culture and non-culture methods [11]. Historically regarded as the “gold standard,” culture methods suffer from low sensitivity, stringent specimen handling requirements, and prolonged processing times, increasing the risk of false negatives and transmission. Nucleic acid amplification tests (NAATs) have revolutionized CT and NG diagnostics due to their superior sensitivity and specificity, largely replacing cell culture methods as the “new expanded gold standard”. However, conventional NAATs, such as PCR and Real-Time PCR, require costly equipment and trained personnel, restricting their use in smaller laboratories and primary healthcare settings [12]. Additionally, delays in result availability following specimen collection can impede patient follow-up [13] – [14]. Hence, there is an urgent need for a sensitive, specific, and user-friendly POCT method [15].

Cross-priming amplification (CPA) is an innovative isothermal DNA amplification technique that has gained attention for its simplicity and efficiency in various diagnostic applications. Unlike traditional PCR methods, CPA does not require thermal cycling, making it suitable for POCT and resource-limited settings. The mechanism of CPA involves the use of a strand displacement DNA polymerase and a set of primers that facilitate the exponential amplification of target DNA under constant isothermal conditions (63 ± 2 °C) [16]. The EasyNAT system, which employs CPA technology, offers rapid and cost-effective diagnostic capabilities and has been effectively utilized in the detection of various pathogens, including *Mycobacterium tuberculosis* [17], *Mycoplasma pneumoniae* [18], *Malaria* [19] and *SARS-CoV-2* [20] with outstanding performance. Currently, there is a lack of research evaluating the performance of the EasyNAT CT/NG assay for detecting CT and NG in clinical settings.

This study aims to evaluate the clinical performance of the EasyNAT CT/NG assay for detecting CT and NG, comparing its efficacy with that of in-house Real-Time PCR, in order to assess its suitability as a rapid POCT method.

## Materials and methods

### Sample collection and preparation

Patients who visited the First Affiliated Hospital of Wenzhou Medical University for CT and NG detection between October 2022 and May 2023 were enrolled in the study. The study was approved by the institutional ethics committee and informed consent was obtained from all participants. Based on preliminary data showing a low positivity rate (~ 15%) in routine clinical testing, an enrichment sampling strategy was implemented to ensure adequate statistical power for diagnostic method comparison. All Real-Time PCR positive samples were included in the study, while Real-Time PCR negative samples were randomly selected to achieve the final study cohort of 198 samples.

For female participants, endocervical swabs, vaginal swabs, and urine specimens were collected; for male participants, urethral swabs and urine specimens were obtained. Each swab (endocervical, vaginal, or urethral) was placed in a tube with 1 mL of sterile saline, and vortexed to release the sample material. Urine samples were centrifuged at 3500 rpm for 5 min, the supernatant was discarded and the remaining 1 mL of sediment. All preprocessed specimens were divided into two aliquots, upon which CT and NG tests were conducted immediately.

### Clinical performance evaluation of the EasyNAT CT/NG assay

Two aliquots of preprocessed specimens were analyzed: one was tested for CT and NG using conventional in-house Real-Time fluorescence PCR assay, while the other was evaluated for CT and NG using the EasyNAT CT/NG assay.

#### Real-time PCR assay

Each sample was analyzed for CT and NG using automated nucleic acid extraction and real-time fluorescence PCR (Anadas9850, AMPLLY Biotechnologies, Xiamen, China) following the manufacturer’s instructions. Post-amplification, the software automatically determined the threshold and detection results based on quality control standards.

#### EasyNAT CT/NG assay

**Fig. 1:**
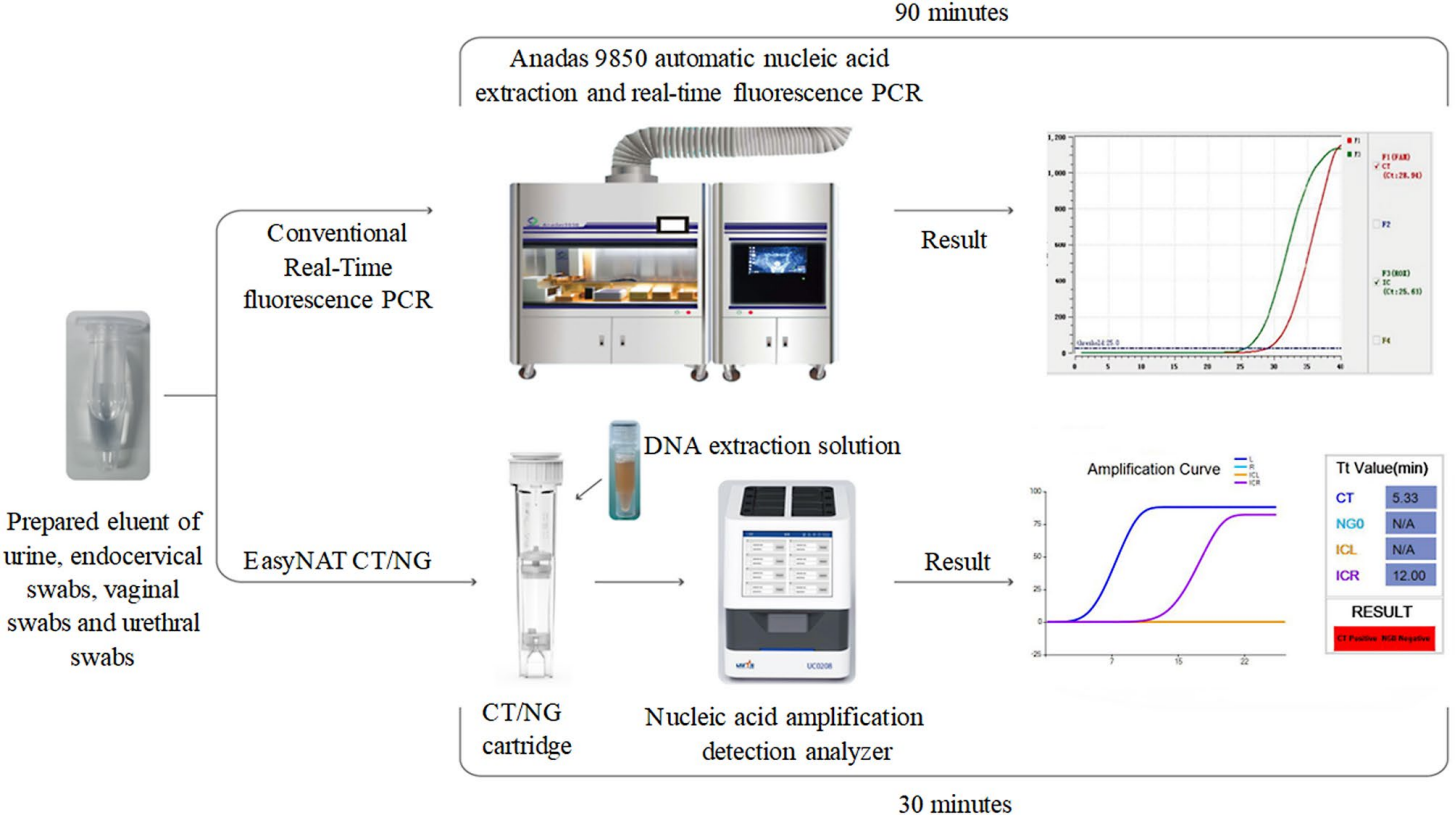
Comparison of Conventional Real-Time PCR and EasyNAT Assays for CT/NG Detection: Workflow and Time Efficiency

The EasyNAT CT/NG assay employs a targeted molecular approach to simultaneously detect CT and NG through amplification of pathogen-specific genetic markers. The assay specifically targets the CDS3 gene for CT detection and the porA pseudogene for NG identification. To ensure assay validity and monitor for potential inhibition or technical failures, an exogenous Bacillus subtilis gene serves as the internal control, providing reliable quality assurance throughout the testing process. All tests were performed strictly according to the manufacturer’s instructions. Briefly, 100 µL of the test sample was added to the cartridge, followed by the addition of the CT/NG DNA extraction solution. The cartridge was then placed in the nucleic acid amplification detection analyzer (UC0208, Ustar Biotechnologies, Hangzhou, China), and the test was initiated by closing the lid and pressing start. Results were automatically displayed and saved upon completion (Fig. 1). The cartridge features multiple hydrophobic layers to separate lysis and cleaning solutions from the reaction solution. Heating by external instruments facilitates chemical disruption and nucleic acid release. Magnetic beads allow nucleic acid samples to move through liquid layers, leading to washing in the cartridge’s leg. This setup allows for fully automated nucleic acid extraction and amplification in a closed system, encompassing lysis, binding, washing, elution and amplification.

### Sensitivity and specificity of the EasyNAT CT/NG assay

#### Sensitivity

To determine the sensitivity of the EasyNAT CT/NG assay, CT and NG bacterial solutions, quantified by digital PCR, were serially diluted to 1600, 800, 400, and 200 copies/mL. For each concentration, 100 µL was added to the cartridge after transferring the entire CT/NG DNA extraction solution. The assay was performed using the nucleic acid amplification detection analyzer (UC0208, Ustar Biotechnologies, Hangzhou, China), with 20 replicates per concentration.

#### Specificity

To evaluate the specificity of the EasyNAT CT/NG assay, 18 common reproductive tract pathogens were diluted to a final concentration of 10^6^ copies/mL separately, as prepared solutions. Separately, CT and NG, quantified by digital PCR, were added to each pathogen solution to achieve 400 copies/mL of CT and NG while maintaining the pathogen at 10^6^ copies/mL. Each mixture was then tested with the EasyNAT CT/NG assay by adding 100 µL to the cartridge after transferring the DNA extraction solution. The analysis was conducted using the nucleic acid amplification detection analyzer (UC0208, Ustar Biotechnologies, Hangzhou, China).

### Evaluation of the EasyNAT CT/NG assay as a POCT

Target Product Profiles (TPPs) criteria provide structured checklists designed to guide both the development and evaluation of STI POCTs. TPPs outline 36 features for POCTs in surveillance, focusing on: (1) Intended Use: Rapid, accurate diagnosis of CT and NG infections for sexually active individuals, high-risk groups, and symptomatic/asymptomatic patients; (2) Performance: ≥90% sensitivity and ≥ 95% specificity; (3) Operational and Usability: Easy to use, room temperature storage, ≥ 12-month shelf life, minimal waste; (4) Cost: < US$ 5, suitable for low- and middle-income countries (LMICs); (5) Quality Standards: Internal controls, external QC compatibility. Detailed criteria will be presented in the “Results” section. This study assessed the EasyNAT CT/NG assay against these TPPs for its suitability as a POCT.

### Statistical analysis

The performance metrics, including sensitivity, specificity, positive coincidence rate, and negative coincidence rate of the EasyNAT CT/NG assay for detecting CT and NG were evaluated using the conventional real-time fluorescence PCR as the reference standard. In cases of discrepancy, the Xpert^®^ CT/NG Assay (Cepheid, Sunnyvale, CA, USA) was used to confirm results. The weighted Kappa (Cohen’s Kappa) statistic was used to assess the agreement between EasyNAT and Real-Time PCR for detecting CT and NG. All statistical analyses were conducted using SPSS Statistics (version 27.0; IBM, USA). Kappa values indicate consistency as follows: <0.2 is poor, 0.2–0.6 is moderate, and 0.6-1.0 is strong.

## Results

### Clinical performance of the EasyNAT CT/NG assay

#### Characteristics of participants and collected samples

This study enrolled 198 participants, including 126 females (63.6%) and 72 males (36.4%), with a mean age of 34.6 years (SD = 11.3); the majority (72.7%) were aged 21–40 years. A total of 198 specimens were collected: 88 endocervical swabs (44.4%), 72 urine samples (36.4%), 28 vaginal swabs (14.1%), and 10 male urethral swabs (5.1%). Specimens were assessed for turbidity and hemolysis. Among urine samples, 39 (54.2%) were turbid and 1 (1.4%) was hemolytic. For endocervical swabs, 26 (29.5%) were turbid and 5 (5.7%) were hemolytic. Vaginal swabs showed turbidity in 11 cases (39.3%), with no hemolysis, while one urethral swab (10%) exhibited turbidity, with no hemolysis observed. Detailed characteristics are provided in Table 1.

**Table 1:** Characteristics of recruitment and collected samples (*n* = 198)

Characteristics of recruitment	*n* (%)
Age (n=198)	
≤ 20	10 (5.1%)
21–30	66 (33.3%)
31–40	78 (39.4%)
41–50	28 (14.1%)
> 50	16 (8.1%)
Gender (n=198)	
Male	72 (36.4%)
Female	126 (63.6%)
Characteristics of Urine specimens (n=72)	
Turbidity	
no	33 (45.8%)
yes	39 (54.2%)
Hemolysis	
no	71 (98.6%)
yes	1 (1.4%)
Characteristics of Endocervical swabs (n=88)	
Turbidity	
no	62 (70.5%)
yes	26 (29.5%)
Hemolysis	
no	83 (94.3%)
yes	5 (6.7%)
Characteristics of Vaginal swabs (n=28)	
Turbidity	
no	17 (60.7%)
yes	11 (39.3%)
Hemolysis	
no	24 (85.7%)
yes	4 (14.3%)
Characteristics of Urethral swabs (n=10)	
Turbidity	
no	9 (90%)
yes	1 (10%)
Hemolysis	
no	10(100%)
yes	0(0.0%)

#### Concordance rates between the EasyNAT CT/NG assay and Real-Time PCR

In this study, 195 samples exhibited concordant CT results, while 3 samples demonstrated discrepant outcomes between the two assays (Kappa = 0.950). Similarly, 196 samples showed consistent NG results, with 2 samples displaying divergent results (Kappa = 0.904). Among the urine samples, 72 provided concurrent NG results, whereas 71 samples reported consistent CT results, and 1 sample yielded a different outcome between the assays (Kappa = 0.972). For endocervical swabs, 86 samples showed concordant CT and NG results, while 2 samples exhibited discrepancies between the assays for both CT (Kappa = 0.913) and NG (Kappa = 0.651). Additionally, 28 vaginal swabs and 10 urethral swabs yielded concurrent CT and NG results across the two assays.

Analysis of 198 clinical samples demonstrated that the EasyNAT CT/NG assay had 100% positive concordance for both CT and NG compared to Real-Time PCR, with negative concordance rates of 98.5% for CT and 99.0% for NG (Table 2). In urine samples (*n* = 72), positive concordance was 100% for both pathogens, with negative concordance of 97.1% for CT and 100% for NG. Among the 88 endocervical swabs, positive concordance remained 100%, while negative concordance was 95.3% for CT and 97.6% for NG. Vaginal swabs (*n* = 28) and urethral swabs (*n* = 10) both showed 100% positive and negative concordance for CT and NG (Table 3). Discrepancies were observed in three samples: one urine specimen (turbid with high crystal concentration) and two endocervical swabs (markedly hemolytic). Retesting with the Cepheid Xpert^®^ CT/NG assay confirmed the urine sample as CT-positive and NG-negative while both endocervical swabs were negative for both pathogens, as detailed in Table 4. The results indicate that urine crystallization did not affect the EasyNAT CT/NG assay whereas it interfered with Real-Time PCR, leading to a missed CT-positive result. However, severe hemolysis adversely affected the performance of the EasyNAT CT/NG assay, causing detection failure.

**Table 2:** Overall performance of CPA compared to Real-Time PCR for detecting *Chlamydia trachomatis* (CT) and *Neisseria gonorrhoeae* (NG) in all specimens (*n* = 198)

	CPA	qPCR		Positive Concordance rates	Negative Concordance rates	Overall Concordance rates
		Positive	Negative	(%)	(%)	(%)
CT	Positive	106^ab^	1^e^	100	96.7	98.5
	Negative	0	89^cd^			
	Invalid	0	2^f^			
NG	Positive	28^ac^	0	100	98.8	99.0
	Negative	0	168^bde^			
	Invalid	0	2^f^			

a, 13 individuals tested positive for both CT and NG with two assays

b, 93 individuals tested positive for CT but negative for NG with two assays

c, 15 individuals tested positive for NG but negative for CT with two assays

d, 74 individuals tested negative for both CT and NG with two assays

e, 1 individual tested negative for CT and NG with Real-Time, but positive for CT and negative for NG with CPA assay

f, 2 individuals tested negative for CT and NG with Real-Time PCR, but invalid for CT and NG with CPA assay

**Table 3:** Performance of CPA compared to Real-Time PCR for detecting *Chlamydia trachomatis* (CT) and *Neisseria gonorrhoeae* (NG) in different sample types

Sample type	Pathogen	CPA	RT-PCR		PC^a^	NC^b^	OC^c^
			Positive	Negative	(%)	(%)	(%)
Urine	CT	Positive	38^g^	1	100	97.1	98.6
(*n* = 72)		Negative	0	33			
	NG	Positive	20^g^	0	100	100	100
		Negative	0	52			
ES^d^	CT	Positive	45^h^	0	100	95.3	97.7
(*n* = 88)		Negative	0	41			
		Invalid	0	2			
	NG	Positive	4^h^	0	100	97.6	97.7
		Negative	0	82			
		Invalid	0	2			
VS^e^	CT	Positive	15^i^	0	100	100	100
(*n* = 28)		Negative	0	13			
	NG	Positive	2^i^	0	100	100	100
		Negative	0	26			
US^f^	CT	Positive	8^j^	0	100	100	100
(*n* = 10)		Negative	0	2			
	NG	Positive	2^j^	0	100	100	100
		Negative	0	8			

^a^PC, positive concordance rates; ^b^NC, negative concordance rates; ^c^OC, overall concordance rates;

^d^ES, endocervical swab; ^e^VS, vaginal swab; ^f^US, urethral swab

g, 7 urine specimens were both positive of CT and NG

h, 3 endocervical swabs were both positive of CT and NG

i, 2 vaginal swabs were both positive of CT and NG

j, 1 urethral swab was both positive of CT and NG

**Table 4:** Xpert^®^ CT/ NG assay for detecting *Chlamydia trachomatis* and *Neisseria gonorrhoeae* of inconsistent cases

			C. trachomatis			*N*. gonorrhoeae		
Cases	Specimen type	Characteristics	Real-Time PCR	CPA	Xpert	Real-Time PCR	CPA	Xpert
Case 31	Urine	Severe turbidity	-	+	+	-	-	-
Case 129	Endocervical swab	Severe hemolysis	-	Invalid*	-	-	Invalid*	-
Case 139	Endocervical swab	Severe hemolysis	-	Invalid*	-	-	Invalid*	-

Invalid*, Inner Control (IC) failed

### Sensitivity and specificity of the EasyNAT CT/NG assay

#### Sensitivity

Sensitivity testing revealed that the 95% confidence interval (CI) for the limit of detection was 332.18 copies/mL for CT and 319.21 copies/mL for NG. Consequently, the analytical sensitivity of the EasyNAT CT/NG assay was set at 400 copies/mL for both CT and NG (Table 5).

**Table 5:** Detection sensitivity of the EasyNAT CT/NG assay

Number of DNA	Positive rate		95% CI (copies/mL)			Lower limit (copies/mL)		Upper limit (copies/mL)	
(copies/mL)	CT	NG	CT		NG	CT	NG	CT	NG
1600	20/20	20/20	322.18	319.21		265.04	254.80	1347.66	3256.34
800	20/20	20/20							
400	20/20	20/20							
200	10/20	11/20							
0	0/20	0/20							

#### Specificity

No cross-reactivity was observed with 18 common reproductive tract pathogens; amplification curves occurred only in the cartridges containing CT or NG DNA (Table 6). These findings indicate the high specificity of the EasyNAT CT/NG assay.

**Table 6:** Detection specificity of the EasyNAT CT/NG assay

Pathogen	Reference Number	Concentration	Results
*Human papilloma virus* 18	ATCC 45152D	10^5^ CFU/mL	CT Negative, NG Negative
*Human papilloma virus* 18 + CT + NG			CT Positive, NG Positive
*Herpes simplex virus* II	ATCC VR540	10^5^ CFU/mL	CT Negative, NG Negative
*Herpes simplex virus* II + CT + NG			CT Positive, NG Positive
*Treponema Pallidum*	ATCC 27,087	10^6^ CFU/mL	CT Negative, NG Negative
*Treponema Pallidum* + CT + NG			CT Positive, NG Positive
*Ureaplasma Urealyticum*	ATCC 33,175	10^6^ CFU/mL	CT Negative, NG Negative
*Ureaplasma Urealyticum* + CT + NG			CT Positive, NG Positive
*Mycoplasma hominis*	ATCC 23,114	10^6^ CFU/mL	CT Negative, NG Negative
*Mycoplasma hominis* + CT + NG			CT Positive, NG Positive
*Mycoplasma genitalium*	ATCC 33,530	10^6^ CFU/mL	CT Negative, NG Negative
*Mycoplasma genitalium* + CT + NG			CT Positive, NG Positive
*Staphylococcus epidermidis*	ATCC 12,228	10^6^ CFU/mL	CT Negative, NG Negative
*Staphylococcus epidermidis* + CT + NG			CT Positive, NG Positive
*Escherichia coli*	ATCC 25,922	10^6^ CFU/mL	CT Negative, NG Negative
*Escherichia coli* + CT + NG			CT Positive, NG Positive
*Gardnerella vaginalis*	ATCC 14,018	10^6^ CFU/mL	CT Negative, NG Negative
*Gardnerella vaginalis* + CT + NG			CT Positive, NG Positive
*Candida albicans*	ATCC 10,231	10^6^ CFU/mL	CT Negative, NG Negative
*Candida albicans* + CT + NG			CT Positive, NG Positive
*Trichomonas vaginalis*	ATCC 30,238	10^6^ CFU/mL	CT Negative, NG Negative
*Trichomonas vaginalis* + CT + NG			CT Positive, NG Positive
*Lactobacillus crispatus*	ATCC 33,820	10^6^ CFU/mL	CT Negative, NG Negative
*Lactobacillus crispatus* + CT + NG			CT Positive, NG Positive
*Adenovirus*	ATCC VR-930	10^5^ CFU/mL	CT Negative, NG Negative
*Adenovirus* + CT + NG			CT Positive, NG Positive
*Human cytomegalovirus*	ATCC VR-977D	10^5^ CFU/mL	CT Negative, NG Negative
*Human cytomegalovirus* + CT + NG			CT Positive, NG Positive
*Beta streptococcus*	ATCC 15,185	10^6^ CFU/mL	CT Negative, NG Negative
*Beta streptococcus* + CT + NG			CT Positive, NG Positive
*Human immunodeficiencyvirus*	ATCC VR-3351SD	10^6^ CFU/mL	CT Negative, NG Negative
*Human immunodeficiencyvirus* + CT + NG			CT Positive, NG Positive
*Lactobacillus casei*	ATCC 393	10^6^ CFU/mL	CT Negative, NG Negative
*Lactobacillus casei* + CT + NG			CT Positive, NG Positive
*Mycoplasma pneumoniae*	ATCC 15,531	10^6^ CFU/mL	CT Negative, NG Negative
*Mycoplasma pneumoniae* + CT + NG			CT Positive, NG Positive

### Evaluation of the EasyNAT CT/NG assay as a POCT

The EasyNAT CT/NG partially meets the TPP criteria for STI POCTs. It achieves optimal sensitivity and specificity for urine, vaginal, and urethral swabs but only meets minimal standards for endocervical swabs. Additionally, the assay exceeds the recommended cost of 5 USD and lacks eco-friendliness due to its single-use cartridge. However, it supports non-invasive sampling, provides data connectivity, and features user-friendly automated preparation with a simple three-step process. It also includes a sample adequacy control for quality assurance. Detailed TPPs compliance is presented in Table 7.

**Table 7:** The EasyNAT CT/NG fulfillment of target products profiles (TPPs) criteria

Characteristics	Criteria	EasyNAT CT/NG assay	Fulfilment
Intended use and target population	Surveillance and case management of sexually active population, screening and regular testing for Key populations and other populations	Screening populations at increased risk of sexually transmitted infections, also applicable for sexually active population	Yes
Target use setting	Health-care settings, especially at primary care level (level 1) or above	Smaller laboratories and primary healthcare settings	Yes
Results	Clear positive, negative or invalid result with minimal instructions for interpretation	Offer clear positive, negative or invalid, easy read	Yes
Equipment	Single use, biodegradable or recyclable disposable diagnostic test preferred, reader optional (small, portable, table-top or handheld, no external electricity or power supply required)	Single cartridge, reader small and portable; but disposal of used materials non-recyclable and power supply required	No
Reference technology	Laboratory-based NAAT	CPA (NAAT based)	Yes
Performance			
Clinical sensitivity Clinical specificity	CT: >90% (minimal) 100% (optimal) NG: 90% (minimal) 98% (optimal) CT: 98% (minimal) 100% (optimal) NG: 90% (minimal) > 98% (optimal)	100% 100% 100% 100%	Yes Yes Yes Yes Yes Yes Yes Yes
Specimen	Vaginal swab or urine (minimal); urine, vaginal, anorectal and oropharyngeal swabs (optimal)	Urine, vaginal, endocervical and urethral	Yes
Specimen preparation	By a health-care provider (minimal); Self-collected samples or by a health-care provider (optional)	Urine samples are self-collected; vaginal, endocervical and urethral swabs are collected by health-care provider	Yes
Specimen collection method	No more than one operator step (minimal); integrated (optimal)	only one operator step	Yes
Steps to be performed between specimen preparation and result	No more than three operator steps that are not timed nor labour intensive (minimal); one operator step (optimal)	3 steps from sample to result	Yes
Internal quality Control-reagents	Quality control internalized in test for each individual test run; positive control available for purchase (minimal); positive control provided in each box of test kits (optimal)	Each reagent kits include the internal quality control	Yes
Cold chain; Test kit stability and storage conditions	None required cold chain at any point; stable between 2 °C and 35 °C, 70% humidity, 3000 m altitude for 12 months (minimal); stable between 0 °C and 50 °C, 90% humidity, 4500 m altitude for 18 months (optimal)	Reagents can be transported between − 25 ℃ and 30 ℃ for 7 days, stored stably for 12 months at 2 ℃ to 8 ℃	Yes
Training required	< 90 min(minimal); 30 min (optimal)	30 min	Yes
Time to result	< 90 min (minimal); 30 min (optimal)	30 min	Yes
Clean water	None	None water required for test running	Yes
Result display and interpretation	Result can be read with the naked eye with minimal instructions, or with an integrated reader with an easy pictorial display	Result display with an integrated reader with an easy pictorial display	Yes
Target price per test	< US$ 5 (minimal); < US$ 1 (optimal)	US$ 9–10 for duplex CT/NG testing	No

## Discussion

POCT for CT and NG has emerged as a critical tool in the diagnosis and management of sexually transmitted infections. POCT enables rapid diagnosis and immediate treatment, which are essential for controlling STI transmission [21] – [22]. The development of NAATs has proven transformative, providing high sensitivity and specificity for detecting CT and NG across diverse specimen types, such as urine, vaginal swabs, oral swabs, rectal swabs, and oral swabs [23] – [24].

In clinical settings, the predominant commercial nucleic acid amplification test (NAAT) platforms employed in the duplex detection of CT and NG are the Cepheid Xpert CT/NG, Aptima Combo 2 and Roche Cobas 4800 CT/NG. These platforms have received FDA approval and demonstrate exceptional sensitivity, ranging from 95.6 to 100%, as well as specificity, ranging from 98 to 100%, for both pathogens [25–28]. We assessed the accuracy of EasyNAT CT/NG assay in clinical specimens, showing concordance rates of 98.4% for CT and 99.4% for NG compared to Real-Time PCR. It performed consistently across specimen types, achieving 100% concordance for CT and NG in urine, vaginal, and urethral swabs, and 96.8% for CT and 98.8% for NG in endocervical swabs. However, like other NAATs, its accuracy depends on sample quality. While turbid specimens did not impair performance, severe hemolysis adversely affected detection, rendering the invalid results.

The Cepheid Xpert, Aptima Combo 2, Roche Cobas 4800, and EasyNAT platforms are capable of detecting a variety of sample types, including urine, vaginal swabs, and cervical swabs. Notably, the Cepheid Xpert and Aptima Combo 2 extend this capability to include rectal and throat swabs, as approved by the Food and Drug Administration (FDA) [28–30]. Recent studies suggest that the Aptima Combo 2 is also capable of detecting frozen semen [31]. This versatility is crucial for conducting comprehensive screenings across diverse patient populations, providing a broader scope of detection compared to the Roche Cobas 4800 and EasyNAT platforms. The Roche Cobas 4800 detects CT/NG in 3.5 h, Cepheid Xpert in 90 min, and Aptima Combo 2 in 3 h [32]. In contrast, EasyNAT only takes 30–45 min using the CPA technique, an isothermal method that eliminates the need for temperature cycling as required in PCR [16]. However, unlike the other three assay, which yield quantitative results, EasyNAT does not provide quantitative measurements for CT and NG. Furthermore, the EasyNAT and Cepheid Xpert’s detection throughput is limited to 16 channels, compared to Roche Cobas’s 96 and Aptima Combo’s 120 [32]. However, unlike the large and costly instruments of Cobas and Aptima Combo, EasyNAT and Cepheid Xpert offer compact and portable devices. Regarding pricing, the Cepheid Xpert test for detecting CT and NG is comparatively costly. In contrast, the price of Roche Cobas 4800 test is approximately 1/3 to 1/5 that of Cepheid Xpert’s [33]. The Aptima Combo 2 test is infrequently utilized in China, and its pricing remains unspecified. Conversely, the cost of the EasyNAT test is lower than that of Roche Cobas 4800’s. Consequently, EasyNAT CT/NG assay are more appropriate for the screening of CT and NG in smaller laboratories and primary healthcare settings.

In 2023, the World Health Organization (WHO) convened global experts to review POCT technologies for STIs including CT and NG, and finally proposed TPPs outlining 36 analytical and operational characteristics for POCTs in surveillance, screening and case management [34]. No test fully met al.l TPP criteria, but the EasyNAT CT/NG assay satisfied most, underscoring its strong potential as a POCT.

In summary, the EasyNAT CT/NG has the following main shortcomings. Primarily, it functions as a qualitative rather than a quantitative test, lacking the capability to quantify DNA and assess the effectiveness of infection treatment. Secondly, the cost of this assay is now slightly above the WHO standard of under USD 5, necessitating a reduction. Additionally, it cannot detect antimicrobial resistance in NG, a critical need given the challenges in developing POCTs for resistance to antibiotics like ceftriaxone and azithromycin [35] – [36]. The experimental design presents certain limitations, notably exhibiting reduced statistical power for CT and NG in urethral swabs, and for NG in endocervical and vaginal swabs, due to small urethral swab sample sizes and low positive NG incidence in endocervical and vaginal swabs. We’ll enlarge the sample size for future verification. Furthermore, the clinical samples that tested positive for CT or NG generally exhibited low Ct (Cycle threshold) values in Real-Time PCR, indicating high levels of CT and NG. Consequently, we did not have enough weakly positive samples to adequately evaluate the clinical performance of EasyNAT CT/NG assay.

## Conclusion

The EasyNAT CT/NG assay exhibits strong concordance with Real-Time PCR in clinical samples. Its simplicity and rapid 30–45 min turnaround time, combined with high sensitivity (detection limit of 400 copies/mL for CT DNA and/or NG DNA) and specificity (no cross-reactivity with other pathogens), make it a suitable POCT for CT and NG detection.

## Data Availability

No datasets were generated or analysed during the current study.
